# Mesoscale and wind-driven intra-annual variability in the East Auckland Current

**DOI:** 10.1038/s41598-021-89222-3

**Published:** 2021-05-07

**Authors:** Rafael Santana, Sutara H. Suanda, Helen Macdonald, Joanne O’Callaghan

**Affiliations:** 1 National Institute of Water and Atmospheric Research, Wellington, 6021 New Zealand; 2 Department of Marine Science, University of Otago, Dunedin, 9014 New Zealand; 3 University of North Carolina Wilmington, Wilmington, NC 28403 USA

**Keywords:** Physical oceanography, Fluid dynamics

## Abstract

Intra-annual variability in the East Auckland Current (EAuC) was studied using a year-long timeseries of in situ and remotely-sensed velocity, temperature and salinity observations. Satellite-derived velocities correlated well ($\hbox {r} > 0.75$) with in situ observations and well-represent the long-term ($> 30$ days) variability of the upper ocean circulation. Four mesoscale eddies were observed during the year (for 260 days) which generated distinct flows between the continental slope and rise. The EAuC dominated the circulation in the continental shelf break, slope and rise for 110 days and generated the most energetic events associated with wind forcing. Current variability on the continental slope was coherent with along-slope wind stress (wind stress curl) at periods between 4 and 12 days (16 and 32 days). We suggest that along-slope winds generated offshore Ekman transport, uplift on the shelf-break, and a downwind geostrophic jet on the slope. In contrast, positive wind stress curl caused convergence of water, downwelling, and increased the current speed in the region. Bottom Ekman transport, generated by the EAuC, was suggested to have caused the largest temperature anomaly ($-1.5 ^{\circ }\hbox {C}$) at the continental shelf-break.

## Introduction

Western boundary currents (WBCs) form a key part of the global climate system by a meridional redistribution of heat^1^. Variability in WBCs occur on timescales of decades^2^, seasons^3^, and days^4^. This variability can be caused by external forcing (e.g., winds), the arrival of remotely-generated eddies, or WBC intrinsic nonlinearity, and the consequent local generation of mesoscale eddies (10–200 km radius)^3,5^. Nonlinear variability in WBCs can be created by horizontal (barotropic) and/or vertical (baroclinic) velocity shear^6^ and promote the formation of mesoscale eddies. These eddies account for more than 50% of the variability in the open ocean ($> 4000$ m depth) with an average lifetime of 8 months^7^. However, near the continental margins, eddies can be relatively short-lived (10 days to 4 months)^8^. Depending on their location and polarity relative to the WBC, mesoscale eddies can increase the magnitude of the WBC flow or generate flow reversals^8^. Eddy variability in WBCs have been observed at periods ranging between 90 and 180 days^3^ and 20 and 45 days^5^ in the East Australian and Brazil Currents, respectively. Local wind stress transfers momentum from the atmosphere to the ocean and, therefore, can transfer energy into the WBC. In addition, along-slope wind stress (AWS) is known to generate Ekman transport which creates a slope in the sea surface height (SSH)^1^. This process also generates a downwind geostrophic current that can alter the WBC speed^9^. A wind stress curl (WSC) close to the continental slope can force convergence, enhancing WBC transport. On the other hand, a negative (positive) WSC can generate divergence and a near-continental-slope flow reversal in the Southern (Northern) hemisphere.

**Figure 1:**
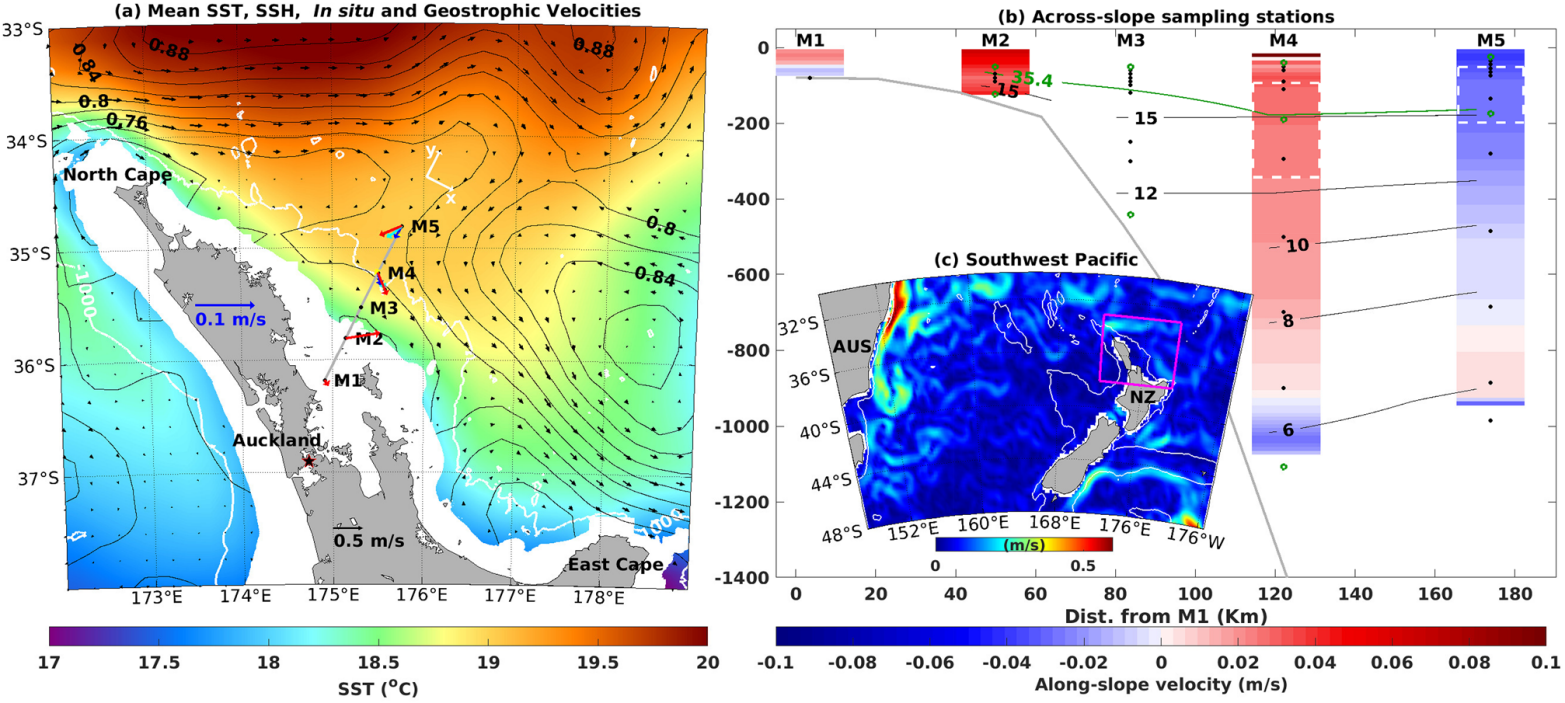
(**a**) Study area showing the average SSH (black contours), geostrophic velocities (black arrows) from AVISO, and sea surface temperature (SST) (shading) from AVHRR. Regions shallower than 200 m are masked (white). The coloured arrows represent, where possible, the vertically averaged (bin $= 280$ m) in situ velocities centered at 140 m (red), 420 m (cyan), 700 m (blue), and 980 m (yellow). The white arrows represent the orientation axes used. The stations M1 to M5 are indicated along the grey line. (**b**) Temporal averages of depth-resolved along-slope (U) velocities (shading), temperature (black contours), and salinity (green contour). The white-dashed rectangles show regions of interpolated velocities between the median depths of the upper ADCP and top bin of the lower ADCP. The vertical positions of thermistors and CTDs are indicated as black dots and green circles, respectively. A deeper CTD is located at M5 in 1864.6 m depth but it is not shown here. (**c**) Location of the study area (magenta contour) relative to other currents in the Southwestern Pacific Ocean. The colors represent the 1-year average of geostrophic speed. The 1000-m isobath is shown as a white contour in (**a**) and (**c**). The maps were generated with m_map (http://www.eos.ubc.ca/~rich/).

The East Auckland Current (EAuC, Fig. 1a) is a unique WBC that originates as the reattachment of subtropical water flow along the continental margin of the New Zealand Northeastern Continental Slope (NZNES)^10^ (Fig. 1c). What is considered the EAuC region is connected by two recurrent eddies: the North Cape Eddy (NCE) and the East Cape Eddy (ECE)^10,11^. The EAuC mean transport was estimated to be 9 Sverdrups (Sv)^11^ with variability at periods longer than 100 days^12^, which can be driven by the arrival of baroclinic Rossby waves (e.g.^13^). Zeldis et al. (2004)^14^ studied the impact of local winds on the EAuC over the continental shelf and found that local winds, through Ekman dynamics, dominate the circulation and water properties. On the inner slope (500 m) the EAuC caused shallow (60 m) intrusions of subtropical water and may be responsible for across-slope flows via bottom Ekman transport (e.g.^15,16^).

Recent work investigated inter-annual variability of EAuC finding that local WSC was correlated ($\hbox {r}=0.43$, ${p}=0.06$) with EAuC transport^17^. An evaluation of the driving mechanism, periods of variability, and possible lags between forcing and ocean response was beyond the resolution of available datasets. Moreover, a possible relation between EAuC transport and AWS has not been evaluated. Stanton and Sutton (2003)^12^ used XBT lines and the altimeter track Topex/Poseidon 071 to suggest that variability in the EAuC is controlled by water volume recirculating in the NCE. The occurrence of topographically controlled and subsurface reverse (NW) flows on the continental slope/rise using half-year time-series of sparse current meters was also evident^10^. A thorough description of the driving processes of EAuC variability is still needed (e.g.^18^).

The goal of this study was to describe the EAuC intra-annual variability and its possible drivers. In this study, we have analysed year-long observations of velocities profiles, temperature, and salinity from five stations across the NZNES (Fig. 1b). The depths of observations were from 80 to 1865 m water depth and span May 2015 to May 2016. The analysis of the moored data combined with remotely-sensed observations of SSH and SST provided a daily picture of the EAuC and mesoscale eddies surface and subsurface conditions. Synoptic near-surface winds were used in combination with the ocean state to better understand wind-driven variability.

## Results

### Satellite observations of the EAuC and mesoscale eddies

**Figure 2:**
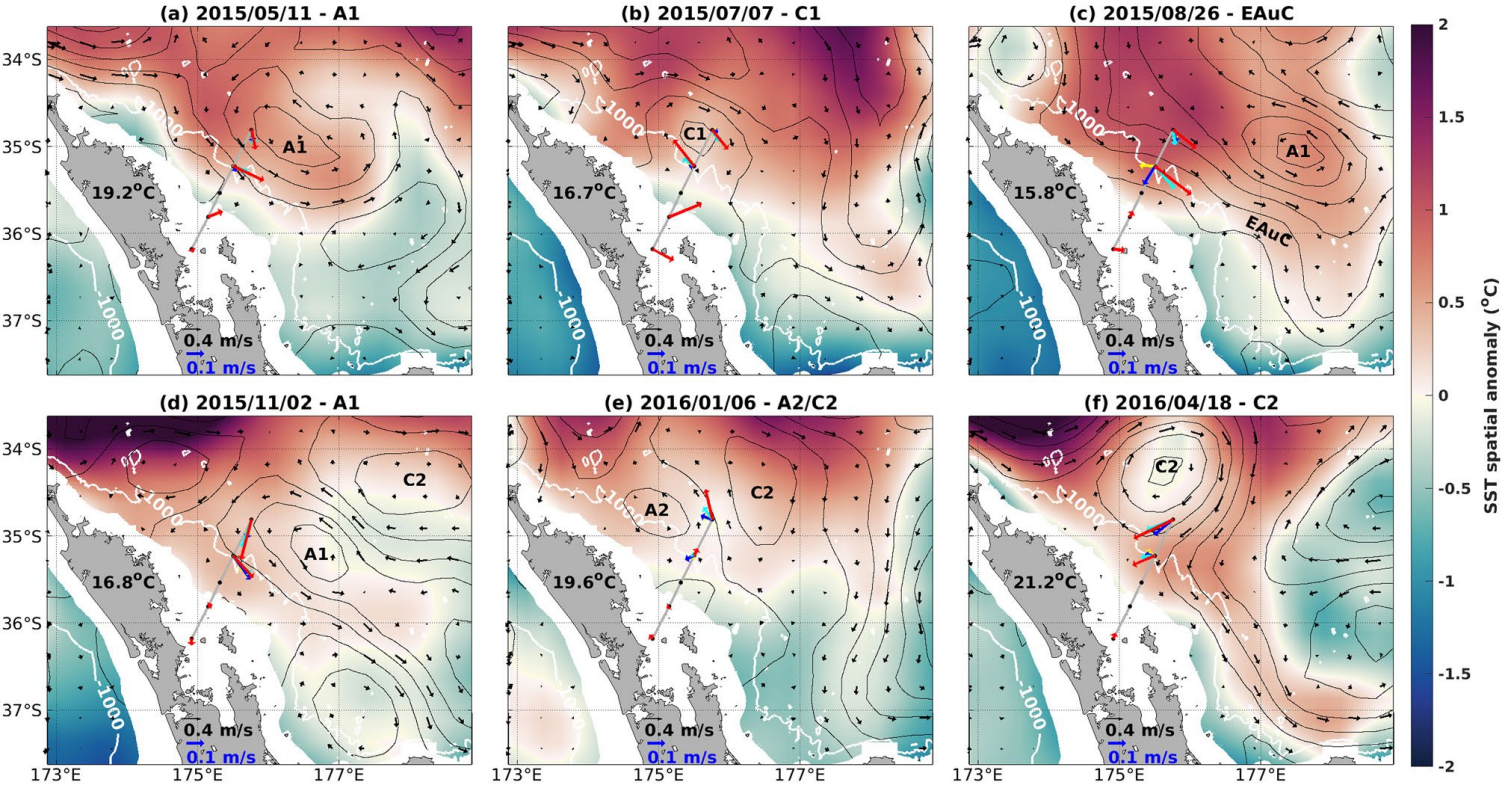
Maps of SST spatial anomaly (coloured shade) (mean value in black), geostrophic (black arrows) and daily averaged in situ velocities (coloured arrows as in Fig. 1a) on (**a**) 11/5/2015, (**b**) 7/7/2015, (**c**) 26/8/2015, (**d**) 2/11/2015, (**e**) 6/1/2016, and (**f**) 18/4/2016 showing the mesoscale structures A1, C1, EAuC, A1, A2/C2, and C2, respectively. The maps were generated with m_map (http://www.eos.ubc.ca/~rich/).

The EAuC has known high interannual variability^17^ and understanding dominant dynamics at the annual timescale (from the year-long measurements) required disentangling mesoscale features that were evident in the moorings observations. Mean circulation showed the core of the EAuC further offshore than the moorings (Fig. 1a), however, daily averages of the flow showed substantial variability and the WBC was present near the slope between early July and mid-December (Fig. 2). Two anti (A1 and A2)—and two cyclonic (C1 and C2) eddies also impacted the moorings during 2015–2016. Initially (May-2015), the southwestern flank of A1 forced a southeastern flow near M4 and M5 (Fig. 2a), advecting warmer waters ($>19.2 ^{\circ }\hbox {C}$) from north of $34.5 ^{\circ }\hbox {S}$ towards the moorings’ region. On 15/6/2015, A1 translated southward and a cyclonic eddy (C1) was evident in the mooring observations. On 7/7, C1 caused a reverse (NW) flow at M4 and a SE flow at M5 (Fig. 2b) and A1 was centered east of the moorings (around $178 ^{\circ }\hbox {E}$ and $35.5 ^{\circ }\hbox {S}$) interacting with a tongue of warm waters on its northern flank (Fig. 2b).

The EAuC was first present at M5 on 16/7, squeezing C1 towards the continental slope. On 26/8, the EAuC dominated flow at M4 and M5 with similar surface magnitudes at both stations (red arrows Fig. 2c). Inspection of depth resolved velocities identified differences between the two stations (blue and yellow arrows in Fig. 2c). At M4, a shoreward component of the velocity and a reverse flow (NW) were seen at 700 m and 980 m respectively. At M5, the flow was unidirectional to the southeast direction. On the same day, A1 (which was located around $177.5 ^{\circ }\hbox {E}$ and $35 ^{\circ }\hbox {S}$) advected towards the moorings, arriving near M5 at the beginning of November 2015 (Fig. 2d). On 2/11, A1 forced along- and across-shore flows at M4 and M5, respectively.

A1 geostrophic velocities persisted until the 9/12, when a dipole formed by an anti- (A2) and a cyclonic eddy (C2) started to dominate the circulation in the region. A2 was locally formed, whereas C2 arrived from the east. Early January, the dipole pushed water northward at M5 and weak currents were observed at M4 and M3 (Fig. 2e). A2 was a smaller eddy (25 km radius) than C2, and became squeezed between the continental slope and C2. C2 grew and dominated the circulation in the region from 10/1 to beyond 15/5/2016. C2 caused weak, moderate, and relatively stronger westward flows at M3, M4 and M5, respectively and reached its maximum size (80 km radius) on 18/4 (Fig. 2f). This cyclonic eddy had strong reverse (NW) flow and was present in 30% of the observation period. C2 (seen in Fig. 2f) strongly influenced the mean circulation (Fig. 1a).

### Mooring time series of the EAuC and eddies

**Figure 3:**
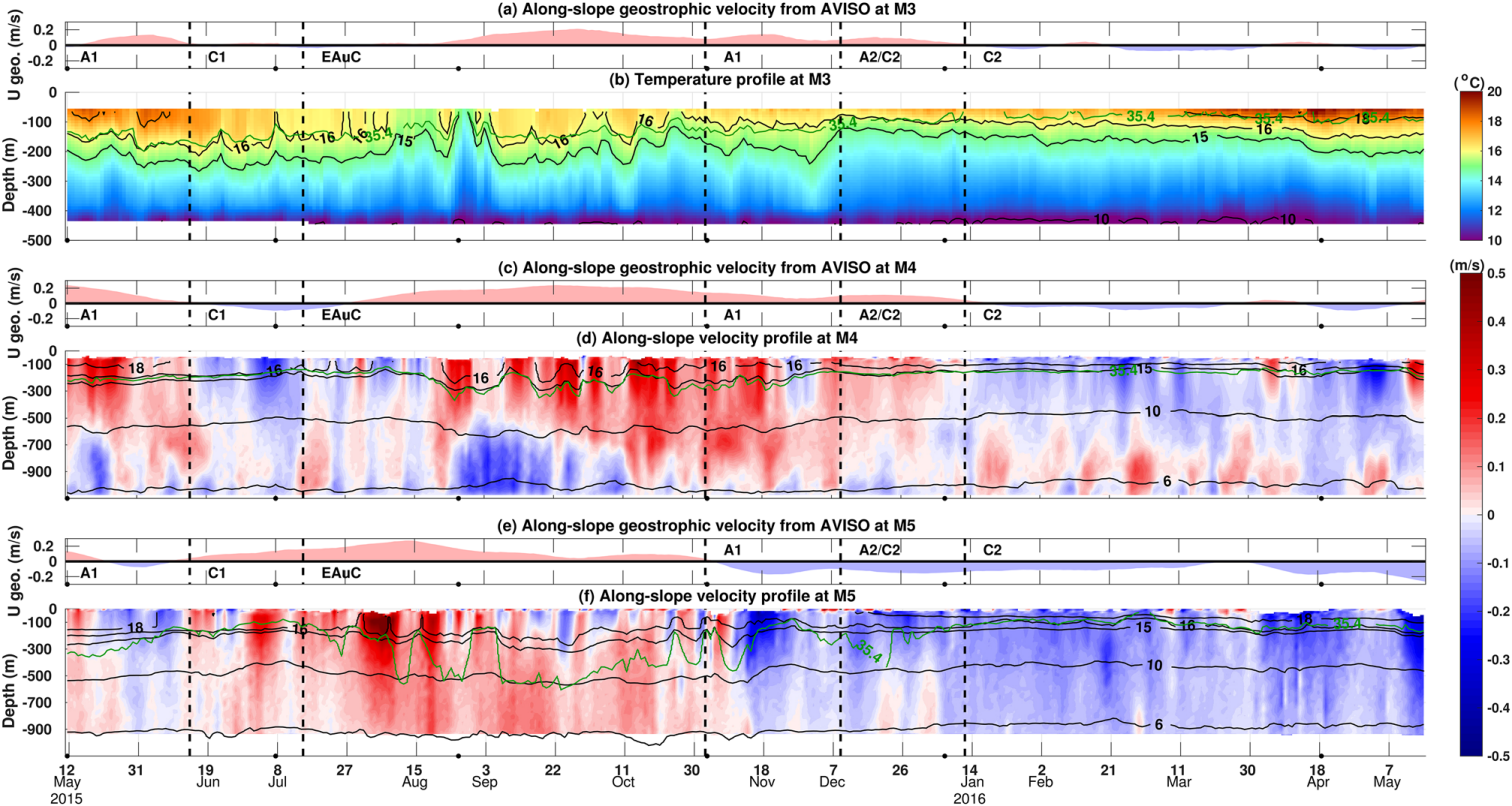
(**a**), (**c**), and (**e**) time series of along-slope geostrophic velocities from AVISO at M3, M4, and M5, respectively. Pink (positive value) indicates a southeastward current. The acronyms A1, C1, EAuC, A1, A2/C2, and C2 represent the presence of mesoscale structures (described in the text) at station M5. (**b**) Temperature daily average profile (shading), (**d**) and (**f**) along-slope daily average of velocities (shading), isotherms (black lines), and 35.4 g/kg isohaline (green line) are shown at M3, M4, and M5, respectively. The black dots mark the dates shown in Fig. 2.

Temporal variability of along-slope velocity, temperature, and salinity at the three offshore stations examined intra-annual dynamics. Mooring time series were segmented according to the eddies and EAuC periods to understand detailed in situ cross-shelf structure of these features (Fig. 3). This analysis showed the distinct variability present in the in situ data compared to satellite observations.

Along-slope (U) (Fig. 1a) geostrophic velocities corresponded well with in situ observations at M4 and M5 (Fig. 3c–f) and were analysed at M3 as well (Fig. 3a). Fluctuations in the direction and magnitude of the flow was dominated by the presence of the EAuC and mesoscale eddies, however, high-frequency pulses in the in situ U velocities were observed, especially when the EAuC was present. Reverse flows at depth were frequently observed at both stations. This reverse flow was stronger and more frequent at M4, where the current tended to change direction around 600 m with the exception of a period between mid-October and mid-November at M4. Several depth-reversal events occurred below 400 m depth at M5 (late May, September, and December, and early January), usually associated with negative flows near the surface. M5 observations are from the upper half of the water column and reversals could be occurring below the measured depth at this station but were not observed.

Increased stratification was observed between early March and early June. An increased near-surface temperature gradient was seen via the presence of the $18 ^{\circ }\hbox {C}$ and $15 ^{\circ }\hbox {C}$ isotherms between 100 and 300 m depth in the initial and final stages of measurements at M3, M4 and M5. Larger temperature variability occurred at 100 m depth where the $16 ^{\circ }\hbox {C}$ isotherm had more vertical oscillation at M3 and M4 in comparison to M5. In late August, the strongest upwelling/uplift event at M3 occurred when the $15 ^{\circ }\hbox {C}$ isotherm was uplifted from 200 to above 50 m depth for the only time at that station. The 35.4 g/kg isohaline tended to follow the $16 ^{\circ }\hbox {C}$ isotherm at M3 and was also uplifted to above 50 m during this event. A similar movement was observed at M4 where the 35.4 g/kg haline marker and the $15 ^{\circ }\hbox {C}$ isotherm had similar positions in the water column ( 200 m). At M5, the 35.4 g/kg isohaline tended to follow the $16 ^{\circ }\hbox {C}$ isotherm during more stratified periods.

### Cross-shelf dynamics during mesoscale events

**Figure 4:**
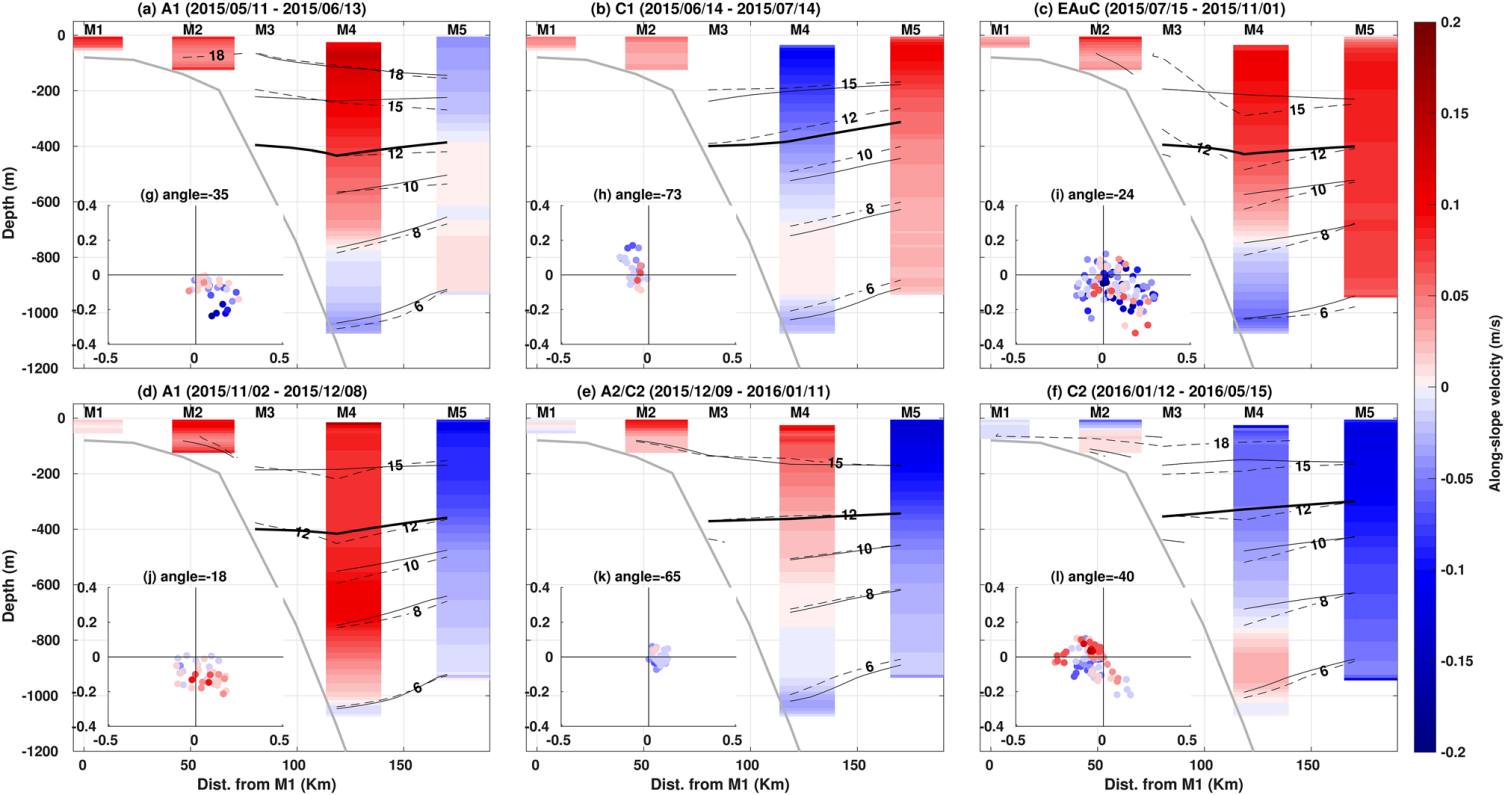
Mean U velocity (red-blue shade) and temperature (solid black line) fields, and daily average temperature (dashed lines) for the mesoscale structures: (**a**) A1, (**b**) C1, (**c**) EAuC, (**d**) return of A1, (**e**) dipole A2/C2, and (**f**) (C2). The daily average temperature fields are from the same days as in Fig. 2: (**a**) 2015/5/11, (**b**) 2015/7/7, (**c**) 2015/8/26, (**d**) 2015/11/2, (**e**) 2016/1/6 (**e**), and 2016/4/18 (**f**). The $12 ^{\circ }\hbox {C}$ is highlighted as a solid bold black line. The hodographs of the upper 270 m depth-averaged water flow (dots) combined with along-slope flow depth-averaged between 840 m and 1120 m depth (red-blue shade equal to the colorbar) for each mesoscale event mentioned in (**g**), (**h**), (**i**), (**j**), (**k**), and (**l**).

The impact of each mesoscale structure on the along-slope velocity and temperature fields across the NZNES was examined. We calculated averages per mesoscale event and compared to the annual and daily means. The EAuC and A1 periods were characterized by downwelled isotherms. In contrast, uplift was observed during the presence of cyclonic eddies. This was evident when comparing the depth of the $12 ^{\circ }\hbox {C}$ isotherm in the year-long mean (Fig. 1) with the mean of each event (bold black lines in Fig. 4). The $12 ^{\circ }\hbox {C}$ isotherm was located near 385 m at M3 and M4, and close to 355 m at M5 for the annual mean. The EAuC and A1 depressed the isotherm towards below 400 m, especially at M4 (Fig. 4a,c,d). Cyclonic eddies caused uplifts of the $12 ^{\circ }\hbox {C}$ isotherm of 40 m (C1) and 90 m (C2) at M5 (Fig. 4b,f). During A2/C2 an uplift of 40 m was observed at M4 as C2 was the dominant feature affecting the mooring stations during the event. At M3, the $12 ^{\circ }\hbox {C}$ isotherm did not experience many changes from the annual mean, except during C2 when an average uplift of 45 m was seen. The daily average surface field of C1 (Fig. 2b) showed small positive SST anomalies, however, in the subsurface uplift was observed in all the isotherms (dashed lines in Fig. 4b). On the contrary, C2 showed characteristics of upwelling at the surface (Fig. 2f) but downwelling at depth (Fig. 4f). These results suggested distinct mesoscale eddies impact at the surface and at depth which can be related to eddy size and its position in relation to the moorings, and atmospheric heat fluxes. Downwelling was observed in all the isotherms analysed during the EAuC and A1 at M4 and M5 (Fig. 4a,c,d). At M3, weak uplift was observed during A1 two passages, however, the strongest uplift occurred when the EAuC encroached on that station (Fig. 3a,b). During this event, the $15 ^{\circ }\hbox {C}$ isotherm was uplifted 100 m at M3 in relation to the EAuC mean (Fig. 4c).

With the exception of the EAuC and C2, all events generated oppositely-directed average flows at M4 compared to M5 (Fig. 4). The cyclonic C2 was large enough that both M4 and M5 were simultaneously influenced by a single side of the eddy. The other eddying events had their centres positioned between the two stations, especially C1 and the return of A1 (Fig. 2), causing the average opposing flows at the 2 sites (Fig. 4a,d,e).

Average reverse flows at depth were observed at M4 in all events except during the second passage of A1. The average U velocities changed direction near 750 m during each event, with exception of C1 (655 m). Hodographs for each mesoscale structure showed that the upper 270 m average flow angle varied between $-18 ^{\circ }$ and $-71 ^{\circ }$ at M4 (Fig. 4g–l). Reverse flows near the bottom (red or blue shading) occurred during most of the events (Fig. 3d) and in all experienced directions (Fig. 4). However, the second passage of A1 was dominated by unidirectional flows independent of the upper flow direction (Fig. 4j).

### Depth-resolved variability and correlations

**Figure 5:**
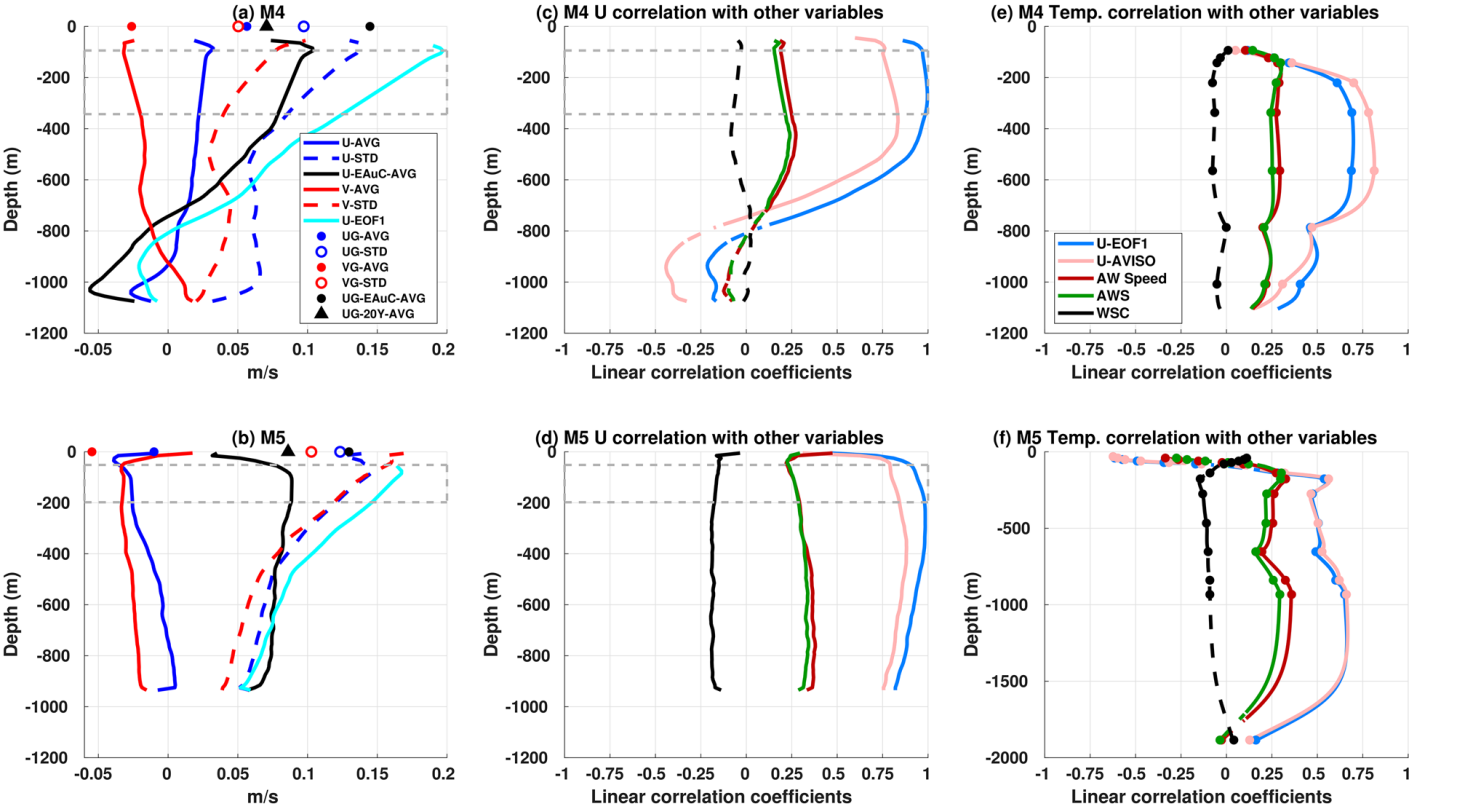
Average and standard deviation of along- (red) and across-slope (blue) velocities at M4 (**a**) and M5 (**b**). The solid and dashed lines represent in situ data results, whereas dots, triangles and circles represent statistics of the geostrophic velocity from AVISO. The black averages are calculated with data from 16/7 to 2/11/2015 (circles and lines) or 1993–2012 (triangles). U-EOF1 (cyan) eigenstructures (non-dimensional) are also shown. The gray-dashed rectangles show the median depths of the upper ADCP and top bin of the lower ADCP. (**c**) and (**d**) depth-resolved linear correlation coefficients between in situ along-slope velocities U-EOF1 (light blue), U-AVISO (pink), along-slope wind speed (dark red), AWS (green), or WSC (black) at M4 and M5. The dots represent the median vertical position of the temperature sensors. Correlations with p value smaller than 0.05 are represented as a dashed line. Similar correlation was applied to temperature (**e**) and (**f**). In (**f**), the depth scale varies from − 2000 to 0 m.

Detailed variability of the velocity field at M4 and M5 were examined in order to identify possible drivers of the along-slope velocity and temperature profiles. Regionally, year-long averages of along- (U) and across-slope (V) velocities at M4 (M5) showed a SSE flow (WSW) (Fig. 5a,b), similar to the surface annual mean (Fig. 1a). Geostrophic velocities from AVISO well-represented the average in situ U component at M5 near the surface and at 350 m. At M4, the V geostrophic component was close to the in situ values from 50 to 200 m depth. However, the geostrophic components overestimated V and U in situ measurements at M5 and at M4, respectively. Average depth-resolved flow tended to have reduced speed from 100 m depth towards the surface at both stations. This feature was clearer at M5 as the Workhorse ADCP was able to measure the currents close to the surface (10 m depth). This phenomenon may be associated with local winds, which might dominate the near-surface average flow over the geostrophic circulation. Standard deviation of the along-slope velocity was approximately two times larger than the across-slope component at M4. Conversely, the variability of the two components of the flow at M5 was very similar with slight differences (0.01 m/s) near the surface and below 600 m depth. Flows along the major axis can be twice to four times greater than the minor axis at M4. However, flows along the major and minor axes have similar magnitudes at M5 when the different mesoscale structures were present.

Reversal with depth was seen in the average circulation at M4, where the along and cross-shore components changed direction at 950 m depth (Fig. 5a). When focusing on depth-resolved and time-average flow during the EAuC period (15/7 to 2/11/2015 - black lines in Fig. 5a,b), it was possible to see a more pronounced reverse flow at 745 m at M4. Significant reduction of surface flows were again present at both stations. Geostrophic velocities from AVISO were overestimated during the same time period. The 20-year average geostrophic velocities had similar values to the upper in situ average during the EAuC time-period for both components. The along-slope velocities first empirical orthogonal function (U-EOF1) explained 71% (91%) of the U component variance at M4 (M5). U-EOFs profiles showed strong surface intensification, however, the decay rate was stronger at M4. This eigenstructure decayed monotonically until 900 m, becoming negative at 800 m, and its values decreased towards zero near the bottom. At M5, U-EOF1 decreased its decay rate at 450 m but did not reach zero nor become negative. As velocities were not measured in the full water column, the eigenstructure may have a different shape with the inclusion of lower water column data.

Along-slope geostrophic velocities and U-EOF1 had similar depth-resolved correlation coefficient patterns with in situ U velocities (Fig. 5c,d). Higher correlation coefficients were found in the upper half of the water column at M4 and M5. Even though the geostrophic velocities were associated with long-term oscillation, the correlation was high ($\hbox {r} > 0.75$) at M4 and M5. Negative correlation coefficients were observed below 755 m at M4. This result was associated with reverse flows at depth that happened throughout the year-long measurements at M4. The correlation decreased sharply above 50 m at both stations, especially at M5 where the lowest value (0.31) was found at 5 m depth. Along-slope winds showed increased correlation values in depths shallower than 40 m at M5. Those results map the depth where the dynamics transition from a friction dominated region to the geostrophic interior. The temperature had moderate (0.5–0.75) correlations with U geostrophic velocities and U-EOF1 between 165 m and 775 m (1635 m) at M4 (M5) (Fig. 5e,f) suggesting that southeastern (northwestern) flows increased (decreased) temperature at those stations. Below 775 m (M4) and 1635 m (M5), the correlation decreased towards 0.15 near the bottom.

### Kinetic energy, winds and U-EOF1 temporal variability

**Figure 6:**
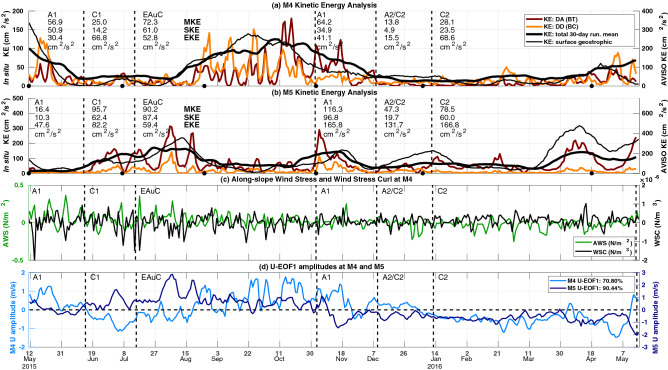
Time series of water column average kinetic energy (KE) divided into depth-averaged (DA) (dark red) and depth-dependent (DD) (orange) at M4 (**a**) and M5 (**b**). The 30-day running mean of the in situ KE (bold black line) and the daily AVISO geostrophic KE (thin black line—right y axis) are also shown. The KE mean (MKE), standard deviation (SKE), and eddy kinetic energy (EKE) for each mesoscale event are also shown. The EKE was computed using the anomalies related to the average from the EAuC period (16/6 to 2/11/2015) (Fig. 3). (**c**) time series of AWS (green) and WSC (black) at M4. (**d**) U-EOF1 amplitude time-series at M4 (light blue— left y axis) and M5 (dark blue—right y axis).

This section studied the temporal variability of the kinectic energy (KE), and its connection with long-term mesoscale variability. Moreover, in situ high-frequency KE variability was seen and linked to along-slope flow which might be driven by the winds. Largest KE occurred when the EAuC was present on the NZNES. The anticyclonic event, A1 also created significant energy peaks at M4 while the other eddying events (C1, return of A1, A2/C2, and C2) generated local KE maxima at M5 (Fig. 6a,b). At M4, the KE was, on average, evenly distributed between depth-dependent KE (53.88%) and depth-averaged KE (46.12%). KE was dominated by the depth-averaged fraction (79.56%) at M5 in comparison to the depth-dependent KE (20.44%). However, this was likely biased towards the depth-averaged fraction as only the top half the water column was measured. At M4 (with full water column measurements), the depth-averaged and depth-dependent represent the barotropic and baroclinic fractions of the KE, respectively. Satellite derived geostrophic KE closely matched trends of the low-pass filtered in situ KE (30-day running mean), with higher energy levels as they only include surface velocity values (Fig. 5a,b). The eddy kinetic energy (EKE) was higher at M4 during the passage of C1 and C2, which caused NW flow opposing the EAuC at this mooring. In contrast, opposing flows at M5 were observed in the second half of the time-series during the return of A1, and the presence of A2/C2 and C2, which caused the highest EKE values. The standard deviation KE (SKE) values per event showed that the EAuC and the first (second) passage of A1 generated the largest variability at M4 (M5). In contrast to high values of MKE and EKE which are associated with the EAuC and eddies, higher SKE is probably generated by other processes (e.g. winds) which may add variability to that generated by mesoscale features.

The AWS (WSC) at M4 has similar variance and is highly correlated ($\hbox {r} >0.9$) with all CCMP wind grid points within 200 (50) km, encompassing both M3 and M5 locations. Therefore, AWS and WSC from M4 will be used in subsequent analysis here. The strongest AWS positive peaks (wind direction coming from NW) occurred early in the record between 12/5/2015 and 3/9/2015, whereas the strongest negative peaks occurred from 26/12 to 18/4/2016 (Fig. 6c). The WSC had its largest variability at the beginning of the measurements to 22/9/2015, after which it reduced until 18/11/2015, and WSC was mostly positive towards the end of the time series. U-EOF1 amplitude time series (Fig. 6d) exhibited similar variability to KE (Fig. 6a,b). U-EOF1 local maxima/minima are often associated with KE peaks at both stations. An exception occurred on 12/7 at M5, when the KE peak was driven by large across-slope velocities.

### Influence of winds

In this section the impact of winds as a forcing mechanism on along-slope velocity and water column temperature (100 m depth) was analysed with wavelet coherency. We identified a range of events and periods with coherent variability and proposed a conceptual diagram of the cross-shelf response to WSC.

**Figure 7:**
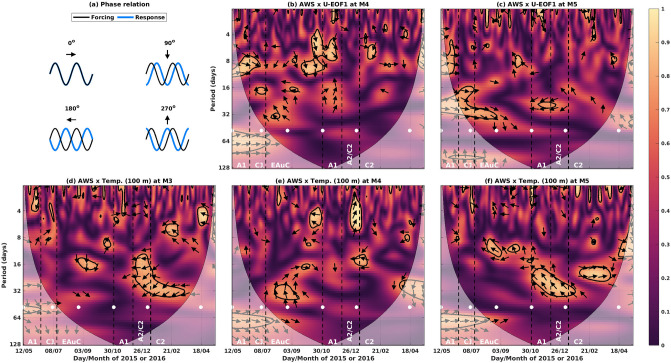
(**a**) Phase relations for wavelet coherency between forcing (black) and response (blue). (**b**) Squared wavelet coherency between AWS (forcing) and U-EOF1 (response) at M4. (**c**) Same as (**b**) but at M5. (**d**) Squared wavelet coherency between AWS and 100 m temperature at M3. (**e**) Same as (**d**) but at M4. (**f**) Same as (**d**) but at M5. The black contour lines indicate coherency above 0.95 at the significance level of $p < 0.05$, and the arrows indicate the relative phase relationship explained in (**a**).

High squared wavelet coherency ($> 0.95$) was found between the AWS and U-EOF1 at M4 and M5 (Fig. 7b,c). At M4, high coherency events occurred at periods between 4 and 12 days. The corresponding phase varies from $0 ^{\circ }$ to $90 ^{\circ }$, meaning that peaks (troughs) in the U-EOF1 occurred between 0 and 4 days after the local maxima (minima) in the AWS (Fig. 7a). At M5, however, high coherency squared was found in longer periods (from 16 to 32 days). The first event with high coherency (from 11/5 to 3/9/2015) had phases closer to zero. In contrast, an anti-phased relationship ($180 ^{\circ }$) was found during the return of A1 and A2/C2 (Fig. 7c). The AWS generates a downwind geostrophic flow which can explain the velocity changes at those stations in their respective periods. The temperature showed high coherency with the AWS in a broad range of periods (4–32 days) among the three stations, and in most cases the phase varied between $180 ^{\circ }$ and $270 ^{\circ }$ (Fig. 7d–f). Positive AWS generates offshore transport via Ekman transport which can explain almost instantaneous ($180 ^{\circ }$), or delayed (one quarter of the cycle, $270 ^{\circ }$) divergence and uplift at 100 m depth. An exception to this occurred during the EAuC at M3 (from 18/8 to 05/10/2015) with phase $90 ^{\circ }$. These results meant an increase in temperature with positive AWS and, therefore, it is unlikely that the AWS was responsible for the strongest uplift event at M3 on 26/8/2015 (Fig. 3b and 4c).

**Figure 8:**
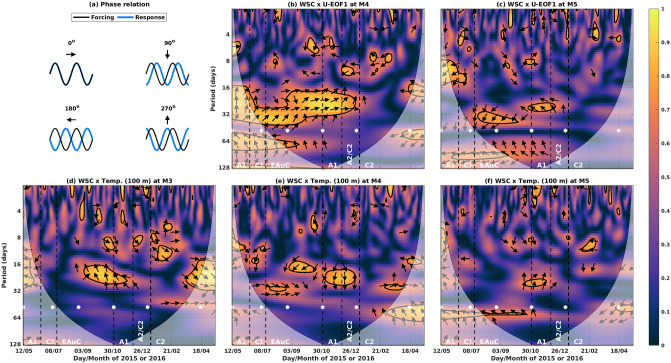
Same as Fig. 7 but for coherency with WSC.

The squared wavelet coherency between WSC and U-EOF1 time-series had a persistent 6-month high coherency event (from 17/6 to 29/12/2015) at M4 (Fig. 8b). The periods of variability with high coherency varied between 16 and 32 days, with phase close to $0 ^{\circ }$ (Fig. 8a) at a period of 30 days. At M5, high coherency was also observed at these periods in distinct events with phases between $135 ^{\circ }$ and $225 ^{\circ }$ (Fig. 8c), in anti-phase with M4 and WSC. Between WSC and temperature at 100 m, high coherency at 30 day period displayed an interesting cross-shore structure. At the inner slope (M3), high coherency was found between 3/9 and 7/12/2015 with phase near $270 ^{\circ }$ (Fig. 8d). At the same time, M4 showed high coherency with phase near $90 ^{\circ }$, 180 degrees out of phase with M3 (Fig 8e). The M5 location also showed high coherency with phase near $270 ^{\circ }$, in phase with M3 (Fig. 8f).

**Figure 9:**
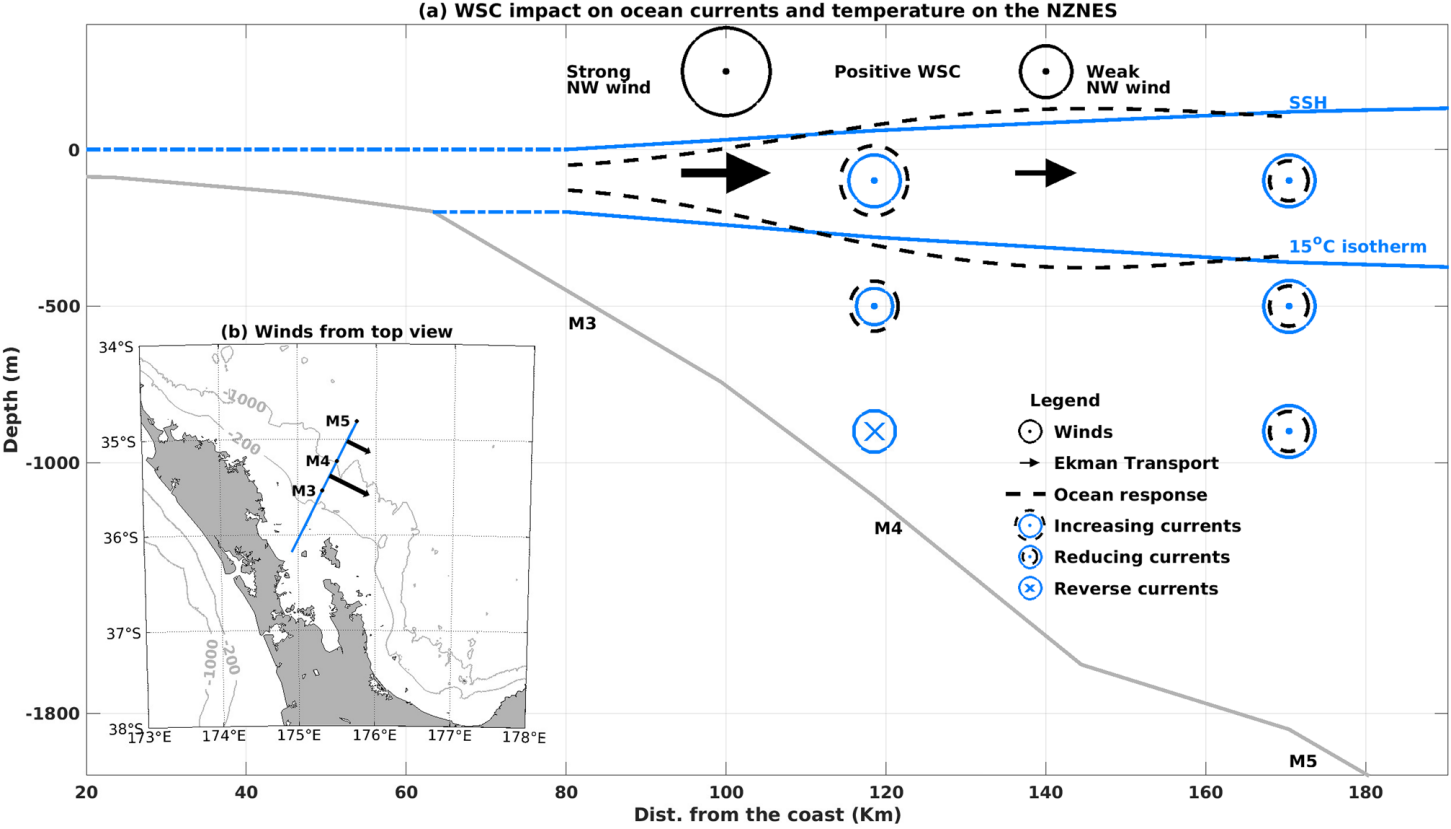
(**a**) Schematic of the WSC forcing on the time mean SSH, $15 ^{\circ }\hbox {C}$ isotherm and currents. The upper (lower) blue line represents the SSH ($15 ^{\circ }\hbox {C}$ isotherm) time mean. The black dashed lines represent the SSH and $15 ^{\circ }\hbox {C}$ isotherm responses to the positive WSC forcing. The dashed blue lines represent regions not studied in detail in the present work. (**b**) Top view of the schematic wind field near the moorings. The map was generated with m_map (http://www.eos.ubc.ca/~rich/).

We propose the following conceptual diagram of the cross-shore response to WSC at 30 day periods. Positive WSC generates a surface convergence at M4, increasing the mean cross-shore SSH slope (dashed surface black line, Fig. 9). This enhances the SE flow magnitude at M4 through the geostrophic relation. At M3, the strong NW wind increases offshore Ekman transport and raises the isotherm at 100 m, out of phase with the drop of that same isotherm at M4 due to the aforementioned convergence. Mooring M5, located further offshore, experiences a relative relaxation of the SSH slope, with rising isotherm at 100 m and a reduction in the surface geostrophic flow (Fig. 9).

## Discussion

Several drivers of varying importance affected circulation and temperature on the NZNES during the year-long period. Background circulation was modulated by the EAuC and mesoscale eddies. Meanwhile, AWS and WSC regulated high frequency variability ($<30$ days) in water column velocity, KE, and temperature that are unresolved by satellite observations. The mesoscale structures (EAuC and eddies) are responsible for temperature changes in the water column via horizontal advection, downwelling and uplift. Southeast (Northwest) flows transport warmer (cooler) water, and EAuC and anticyclones or cyclones generate down- or uplift via convergence or divergence of water masses. Mesoscale eddies were evident at the mooring sites for 260 days of the year-long observation period. Both anticyclonic and cyclonic eddies were captured across the mooring transect (Figs. 2 and 4). Mesoscale variability have been documented near North Cape^12^. In our measurements, an anticyclone (A1) originated upstream and propagated southwards past the mooring array. When A1 was positioned just north of East Cape, it would be indistinguishable from what is locally referred to as the ECE. Later, both A1 and a separate cyclonic eddy (C2) arrived at the moorings from the east. At midlatitudes, eddies travel at speeds comparable to Rossby waves, and these features can be similar to those found by Chiswell (2001)^13^ in the region.

Different variability in the flow direction was observed at the continental slope (M4) and rise (M5). At M4, flow was constrained by the continental slope, whereas at M5 the circulation did not show a well-defined direction, even with the presence of the EAuC. Topographically controlled flows, such as the one seen at M4, have previously been found on the slope close to the North Cape^12^. In addition, opposing (NW) flows at depth were found near the North Cape by the same authors^12^. Reverse flows at depth were observed in all oceanic events at M4 except during the return of A1. One fifth of the water column at M4 flowed in an opposite direction in comparison to the surface flow in the whole time-series average. During the EAuC, the lowest quarter of the water-column flowed northwest at a speed of half that of the near-surface magnitude. This is similar to that seen in the EAC region where weak opposing flows were observed below 1500 m^19^. These results suggested that volume transport estimated from altimeter measurements should be taken with care when considering the EAuC extending to the bottom^12,17^. Whilst geostrophic velocity estimates have their limitations, they adequately represented long term variability of the upper-half water column on the continental slope (M4) and rise (M5), answering Wunsch’s (1997)^20^ main question: “given the surface geostrophic velocity as measured by an altimeter, how is the motion to be interpreted as a function of depth?” for the studied region. Apparent differences in velocity profiles between M4 and M5 could be an artefact of the sampling regime or process-related due to topographic controls.

In this work, coherency between in situ water column currents and temperature and AWS and WSC have been documented. The dynamics responsible for the variable response between mooring locations remains to be understood. AWS had high coherency with along-slope circulation at periods $< 12$ days at M4, while at M5 (the most seaward site), the dominant period was longer (16–32 days). The AWS generates a downwind geostrophic flow via Ekman dynamics which can explain the velocity changes in these regions. However, the difference in driving periodicity at the two stations is still to be fully resolved. Positive WSC at M4 can promote water mass convergence on the continental slope which might be responsible for the generation of a geostrophic jet and downwelling at M4 (Fig. 9) at periods between 16 and 32 days. M3 and M5 seemed to be at the border of the pumped waters and showed eventual uplift in correspondence with positive WSC (Fig. 9) at similar periods. The strong uplift (anomaly equal to $-1.5{ ^\circ }\hbox {C}$) on 26/08/2015 at M3 (Fig. 3b) did not coincide with, nor was preceded by, northwesterly winds or with negative WSC. With the arrival of the EAuC, shoreward-directed bottom Ekman transport, could have occurred on the slope (blue arrow in Fig. 4c) and delivered colder water to the continental shelf break (M3) (Fig. 2c). A similar process was suggested by Zeldis et al. (2004)^14^ to be responsible for the arrival of colder water on the continental inner slope (500 m depth).

## Methods

Five cross-shelf oceanographic moorings (M1–M5) were deployed along Topex/Poseidon 147 line in water depths spanning 80 to 1865 m (Fig. 1a). The stations, M1 (80 m depth) and M2 (130 m) were on the continental shelf, M3 (438 m) the shelf break, M4 (1105 m) the continental slope, and the most offshore M5 (1865 m) on the continental rise. The deployments lasted longer than a year and the measurements overlap between 11/5/2015 and 15/5/2016. A total of 46 instruments were installed in the array of moorings (Fig. 1). Long Range (LR) acoustic Doppler current profilers (ADCPs) (75Hz) were deployed at stations M3, M4, and M5, and 4 Workhorse (WH) ADCPs (300 or 600Hz) were installed near the bottom at M1 and M2, and near the surface at M4 and M5 and covered the water column shown in red and blue in Fig. 1b. The water column was binned every 4 m (WH) or 15 m (LR) by upward looking ADCPs. All the stations, except M1, had between 4 and 10 SBE 56 temperature sensors (black dots in Fig. 1b) and 2 or 3 MicroCAT CTDs (green circles in Fig. 1b). The temperature sensors were deployed every 10 m in the upper 100 m and then every 50 m below that. M2 and M3 had CTDs placed near the surface and bottom, whereas M4 and M5 had additional CTD at 200 m depth. Velocity (temperature and salinity) measurements were taken during a period of 2 min. (1 min.) every 10 min or less. The data is freely available and can be found on O’Callaghan et al. (2015)^21^.

Remotely-sensed observations of SSH, SST, and winds were used. Optimally interpolated gridded maps of SSH with $1/4 ^{\circ }$ of horizontal resolution created from AVISO^22^ were used to determine the presence of the EAuC and mesoscale features near the moorings. In addition to the SSH, AVISO also provides derived geostrophic velocities which were compared with the in situ velocity measurements. Daily maps of SST ($1/20 ^{\circ }$) from AVHRR Pathfinder^23^ were used and compared with near-surface in situ measurements. Surface wind vectors from CCMP^24^ were used to determine the wind impact on the EAuC. The final wind speed data was delivered as 6-h maps of $0.25 ^{\circ }$ of resolution which were converted to AWS and WSC following Kampf and Chapman (2016)^9^ and daily averaged.

All in situ data were quality controlled following the standards of the Global Temperature and Salinity Pilot Program from the Intergovernmental Oceanographic Commission^25^ and from the database of the American National Oceanographic Data Center. Principal axes^26^ were computed using daily- and depth-averaged velocity measurements to determine the dominant flow direction at M4 ($32.00 ^{\circ }$) and M5 ($1.96 ^{\circ }$). The major axis was aligned along the local bathymetric slope. Horizontal velocity components were rotated to a coordinate system oriented in the along-(U) and across-slope (V) directions (Fig. 1a). Kinetic energy ($\hbox {KE}=0.5$($\hbox {U}{^2}+\hbox {V}{^2}$)) was analysed in the continental slope (M4) and rise (M5). KE was separated into depth-averaged and depth-dependent components. The depth-averaged component was calculated as the vertical average of the daily velocity profile and the depth-dependent component was calculated as profiles of anomalies from the vertically averaged time series. The EKE was calculated using the daily anomalies obtained with the temporal average during a subset of time when the EAuC was present in the time-series (15/07/2015-2/11/2016). Standard deviation (SKE) of the total KE was computed for both stations (Fig. 6). Empirical orthogonal functions (EOFs)^26^ were used to investigate the variability in time-series of the velocity profiles at M4 and M5. Linear correlation was used to calculate depth-resolved correlation coefficient between ocean state variables (such as velocities/temperature) and probable forcing mechanisms (AWS, WSC, AVISO geostrophic velocity etc). Squared wavelet coherency^27^ was applied between AWS/WSC and velocities/temperature to evaluate their common variability and relative phase in time-frequency space. Coherency was considered valid over a confidence level of 95%.
